# Rat liver ECM incorporated into electrospun polycaprolactone scaffolds as a platform for hepatocyte culture

**DOI:** 10.1002/jbm.b.35115

**Published:** 2022-06-23

**Authors:** Thomas S. R. Bate, William Shanahan, Joseph P. Casillo, Rhiannon Grant, Stuart J. Forbes, Anthony Callanan

**Affiliations:** ^1^ School of Engineering Institute for Bioengineering, University of Edinburgh Edinburgh UK; ^2^ School of GeoSciences University of Edinburgh, Grant Institute Edinburgh UK; ^3^ MERLN Institute Maastricht University Maastricht The Netherlands; ^4^ Centre for Regenerative Medicine University of Edinburgh Edinburgh UK

## Abstract

Liver disease is expanding across the globe; however, health‐care systems still lack approved pharmaceutical treatment strategies to mitigate potential liver failures. Organ transplantation is the only treatment for liver failure and with increasing cases of liver disease, transplant programs increasingly cannot provide timely transplant availability for all patients. The development of pharmaceutical mitigation strategies is clearly necessary and methods to improve drug development processes are considered vital for this purpose. Herein, we present a methodology for incorporating whole organ decellularised rat liver ECM (rLECM) into polycaprolactone (PCL) electrospun scaffolds with the aim of producing biologically relevant liver tissue models. rLECM PCL scaffolds have been produced with 5 w/w% and 10 w/w% rLECM:PCL and were analyzed by SEM imaging, tensile mechanical analyses and FTIR spectroscopy. The hepatocellular carcinoma cell line, HepG2, was cultured upon the scaffolds for 14 days and were analyzed through cell viability assay, DNA quantification, albumin quantification, immunohistochemistry, and RT‐qPCR gene expression analysis. Results showed significant increases in proliferative activity of HepG2 on rLECM containing scaffolds alongside maintained key gene expression. This study confirms that rLECM can be utilized to modulate the bioactivity of electrospun PCL scaffolds and has the potential to produce electrospun scaffolds suitable for enhanced hepatocyte cultures and in‐vitro liver tissue models.

## 1 INTRODUCTION

The burden of liver disease on people and health systems is increasing globally year‐on‐year and estimates now state that approximately 25% of the global population will be showing signs of onset or fully fledged NAFLD.^1^, ^2^, ^3^ This puts a quarter of the world at risk of fibrosis and hepatocellular carcinoma where currently no approved pharmaceutical treatment exists for any stage of liver disease and the eventuality of liver failure brings death without transplant liver availability. Thus, alternative strategies must be explored for the progression of drug development methodologies and other potential treatment methodologies for liver disease. Progressions in 3D cell culture methods have delivered more realistic models of liver tissues in‐vitro, both structurally and functionally.^4^ Culturing cells within a 3D matrix that recapitulates complex in‐vivo cell–cell and cell–matrix interactions allows for more accurate modeling of in‐vivo cell behaviors.

Electrospun scaffolds provide a fibrous polymeric matrix like that of the in‐vivo extra‐cellular matrix (ECM) upon which cells can be attached in‐vitro.^5^ This realistic biomechanical environment entails a simple fabrication process and research has proven its potential in the culture of hepatic cell types for *in‐vitro* models and regenerative cell therapy purposes.^6^, ^7^, ^8^, ^9^, ^10^, ^11^ The morphology of electrospun fibers can be tailored to alter the local mechanical environment upon which cells attach and this has shown the affect the behavior and function of hepatocytes on electrospun polycaprolactone (PCL) scaffolds.^12^ Moreover, many studies have shown that the local mechanics of cell attachment substrates is proven to direct differentiation of both hepatocytes and stem cells.^13^, ^14^, ^15^, ^16^ The bulk of many electrospun scaffolds is generally fabricated from biocompatible synthetic or natural polymers. While these materials can provide a consistent structure with cell attachment motifs, these systems lack the specific biochemical niche that is present within the in‐vivo ECM.

Hepatocytes rely on a dynamic relationship with a particular set of ECM proteins which governs the function of hepatocytes in‐vivo and facilitates communication between neighboring cells and the immune system.^17^, ^18^ ECM proteins such as Collagen, Laminin and Fibronectin can be incorporated into electrospun polymer scaffolds, introducing cell‐matrix interactions that improve cell attachment and even direct cell function and differentiation.^8^, ^19^, ^20^ However, this does not capture the complexity present within native ECM which is composed of hundreds of different components categorized as Collagens, ECM glycoproteins, Proteoglycans, ECM regulators, ECM affiliated proteins and secreted factors. Introducing this complexity into hepatocyte scaffolds can be achieved by stripping cells from dissected liver tissues and incorporating the decellularised liver ECM (dLECM) into scaffold structures.^21^ Introduction of the complex combination of ECM proteins into liver tissue scaffolds has proven to drive altered functional responses from hepatocytes.^9^

Many studies have explored the possibilities of using dLECM for the culture of hepatocytes and have shown that dLECM from caprine, murine, porcine and human sources can support hepatocytes *in‐vitro*.^22^, ^23^, ^24^, ^25^, ^26^, ^27^ Removal of cells and residual cytoplasmic and nucleic materials can be achieved through different chemical and biological methods including detergent exposure, hypertonic‐hypotonic processes, acids and bases and enzymatic treatments such as with trypsin and nucleases.^28^ These methods can be complemented by mechanical removal of the cytoplasmic and nucleic materials such as by mechanical agitation or by perfusion methods. Whole organ perfusion methods have been established in order to retain the entire ECM structure of whole organs in view of repopulating this structure with cells and producing transplantable organs *in‐vitro*.^29^, ^30^ Whole organ perfusion methods have been optimized for cellular material removal and ECM protein preservation in detergent‐based decellularisation of renal tissues.^31^, ^32^ Thus, dLECM obtained via whole organ perfusion also presents a valuable potential resource to explore for the development of dLECM enhanced electrospun scaffolds.

Herein, we report the fabrication of rat‐dLECM (rLECM):PCL electrospun scaffolds for the culture of hepatocytes in‐vitro. Harvested rat livers have been decellularised by vascular perfusion of sodium dodecyl sulfate (SDS) solution. rLECM:PCL scaffolds containing 5 and 10 w/w% rLECM are produced by electrospinning, maintaining morphological consistency between each of the scaffolds. The HepG2 human hepatic carcinoma cell line has been cultured upon the scaffolds and analyzed for cell viability, DNA quantitation, immunohistochemistry, albumin secretion and RT‐qPCR gene expression analysis of key hepatic functional and ECM markers.

## 2 METHODS

### 2.1 Rat liver harvesting and decellularisation

**FIGURE 1:**
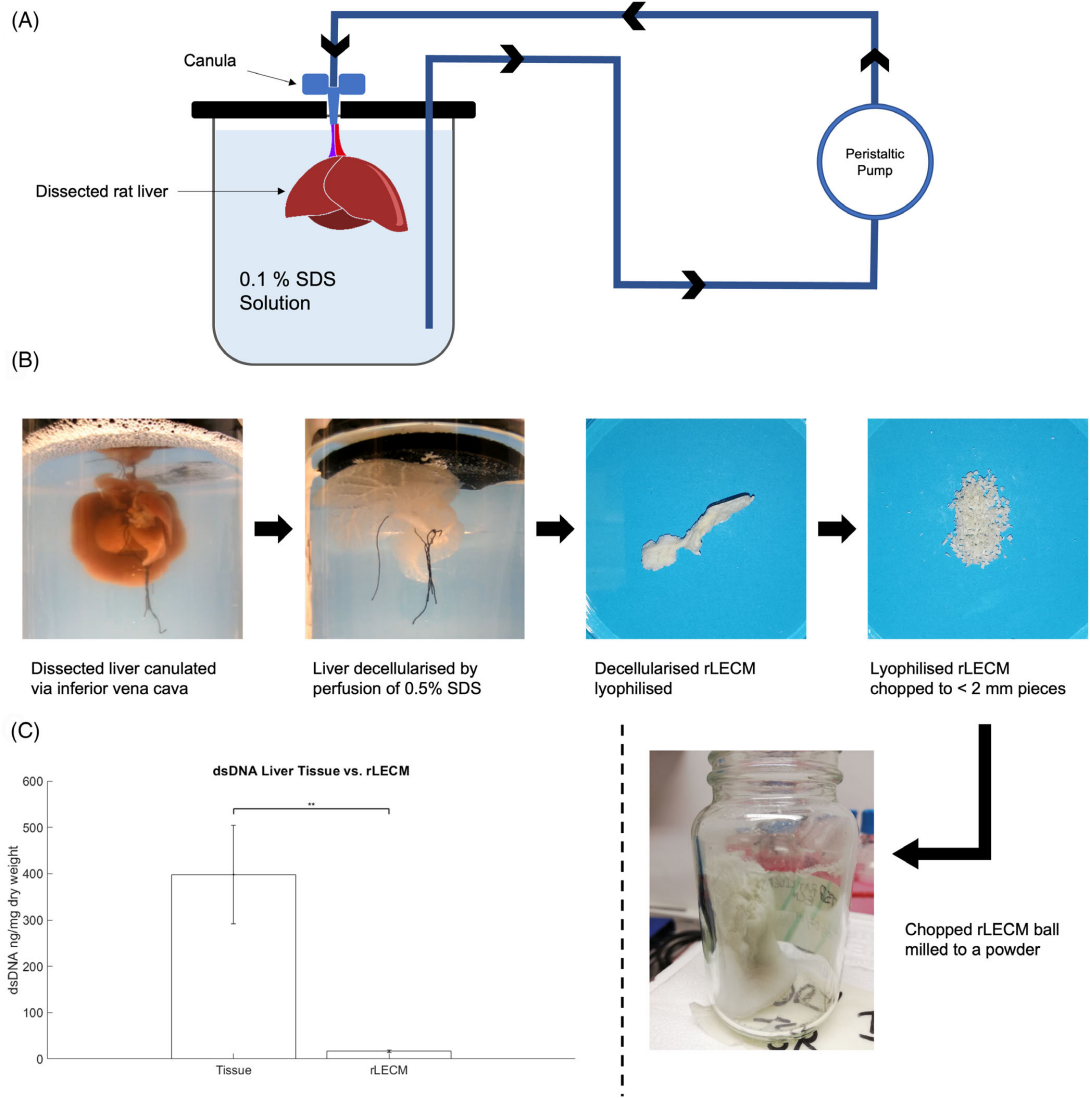
(A) Schematic diagram of the perfusion decellularisation process. (B) Images showing each stage of the rLECM processing featuring decellularisation, lyophilisation, chopping, and ball milling. (C) Graph showing the dsDNA contents of lyophilised tissue and rLECM. *N* = 3, Error bars = SD, One‐way ANOVA results shown with Tukey's post‐hoc test, **p < .01

Rat livers were harvested from 8‐week‐old Sprague Dawley rats immediately after cervical dislocation. Dissected livers were cannulated via the inferior vena cava and perfused initially with 30 ml 10 μg/mL sodium nitroprusside solution to dilate the blood vessels. The liver was then washed by perfusion with 50 ml phosphate buffered saline (PBS) solution. Decellularisation was initiated by connecting the cannula in‐line with a peristaltic pump and the liver submerged in and perfused with 400 ml of 0.1% SDS solution at a flow rate of 10 ml/min, see Figure 1 for a diagrammatical explanation. After 4 h, the SDS solution was changed to a fresh solution and perfusion continued for 16 h before washing with distilled water by perfusion for the same time periods as SDS. Decellularised and washed liver ECM was then stored at −80 °C until lyophilisation using a Labconco freeze‐drying apparatus. After lyophilisation the rLECM was chopped using a scalpel and ball milled to a powder using a Retsch planetary ball mill in 4‐minute cycles. The chamber was cooled with dry ice for 10 min in between a total of 8 cycles.

All experiments with animals were approved by the University of Edinburgh Animal Welfare and Ethical Review Body and the U.K. Home Office. All experiments with animals took place at the Ann Walker Animal Facility, Kings Buidings, Edinburgh and were performed according to the procedural guidelines and severity protocols from the U.K. Home Office Animals Scientific Procedures Act.

### 2.2 Electrospinning and scaffold preparation

Electrospinning solutions were prepared with polycaprolactone Avg. MW = 80,000 (Sigma), hexafluoroisopropanol (HFIP, Manchester Organics) and rLECM. Three scaffold groups were prepared containing 0 w/w% (Control group), 5 w/w% and 10 w/w% rLECM:PCL. rLECM scaffolds were prepared by dissolving 40 mg (5 w/w%) and 80 mg (10 w/w%) of rLECM powder in 10 ml of HFIP. After a brief agitation, 0.8 g of PCL was added to the rLECM:HFIP mixture and the solution was left to fully dissolve on a roller overnight. The solutions were then loaded into a syringe and electrospun as 10 cm wide sheets onto a rotating mandrel with an IME EC‐DIG Electrospinning apparatus. The parameters for electrospinning were as follows: needle diameter = 0.4 mm, flow rate = 1.5 ml/h, needle‐mandrel distance = 15 cm, mandrel rotation = 250 RPM, transverse needle movement: 10 cm width at 5 cm/s. Once electrospun, 12 mm round scaffolds were punched from the sheet material and stored at 4 °C until seeding with cells.

Prior to seeding the scaffolds were washed three times in 70% ethanol and lyophilised in order to sterilize the scaffolds. Post sterilization the scaffolds were submerged in PBS with 1% Anti‐Anti (Gibco) to maintain sterility before seeding.

### 2.3 Scanning electron microscopy

Scaffolds were imaged using a Hitachi HT4000 Plus Scanning Electron Microscope with an accelerating voltage of 15 kV using the mixed sensor mode combining back‐scatter and the secondary electron sensors.

### 2.4 Mechanical testing

Tensile properties of the electrospun fiber materials were assessed using an Instron 3367 tensile testing apparatus. 5 by 1 cm samples were cut from the electrospun sheets using a scalpel. The samples were clamped 1 cm at each end within the tensile testing apparatus giving a gauge length of 3 cm for each sample. Samples were extended at 15 mm/min or 50% strain/min until failure. Calculation of the Young's Modulus at different strain bands was conducted using the MATLAB. The slope of a linear regression formed on the non‐linear stress–strain graph between the chosen strain points was used to estimate the Young's Modulus (see Supplementary Figure 1 for details). Tests were repeated *N* = 5 times.

### 2.5 FTIR and CN elemental analysis

Fourier‐Transform Infra‐Red (FTIR) analysis was conducted on hLECM samples using a Nicolet I10 FTIR apparatus (Thermo) with an Attenuated Total Internal Reflection (ATR) stage. Briefly, small sections of the hLECM samples were cut using a scalpel. After measuring the atmosphere for a blank measurement, sample sections were then loaded onto the ATR stage and clamped into place, ensuring coverage of the stage. Absorbance measurements were then taken at sequential wavenumbers from 4000 to 500 cm^−1^.

For CN analysis, approximately 3–5 mg of PCL fibers, 5% rLECM:PCL fibers and 10% rLECM:PCL fibers were each weighed in triplicate into pressed 8 × 5 mm tin capsules (Elemtex) on a Sartorius SC2 Ultra‐Microbalance. The samples were analyzed on a Thermo Fisher Scientific FlashSmart Elemental Analyzer (Serial # 2018.FLS0037), equipped with a CN/CHN Prepacked Quartz Reaction Tube (Elemtex) and a 2 m stainless steel CN/CHN Separation Column (Elemental Microanalysis). The system was calibrated for carbon and nitrogen using four preparations, ranging from approximately 1–4 mg, of Atropine reference standard (Elemtex) and approximately 2 mg of BBOT reference standard (Elemtex) was used as the calibration check.

### 2.6 Cell culture and scaffold seeding

Cryopreserved HepG2 (Sigma) were raised in T75 flasks (greiner) in complete media containing Minimum Essential Media (MEM, Gibco) with 10% Fetal Bovine Serum (FBS, GE Healthcare), 5% L‐Glutamine 200 mM (Gibco), 5% Non‐essential Amino Acids (NEAA, Sigma) and 5% Penicillin–Streptomycin 10,000 U/mL (Gibco). The cells were expanded in the T75 flask in preparation for seeding the scaffolds, once 80% confluency was reached the cells were trypsinised via standard methods and suspended in complete media. The cells were counted using a hemocytometer and the cell concentration within the suspension adjusted accordingly by addition of complete media. A total of 50,000 cells within 50 μl of complete media were seeded on each scaffold and the cell left to attach in an incubator at 37°C and 5% CO_2_ for 2 h. After cell attachment, 300 μl of complete media was added to the well and media was changed every 3 days for the duration of the study.

### 2.7 Cell viability analysis

Cell viability was assessed using the cell titer blue assay (Promega) which relies on the conversion of resazurin to resorufin within the cell. The assay was conducted on N = 5 samples per group according to the manufacturer's instructions and the fluorescence values were measured on a Modulus II Microplate reader at ex 525 nm/em 580–620 nm.

### 2.8 DNA quantitation

All scaffold groups (N = 5 samples per group), 5 mg of lyophilised tissue and 5 mg of rLECM were placed in Papain digest solution containing 2.5 U Papain, 5 mM Cysteine‐hydrochloride (Sigma) and 5 mM Ethylenediaminetetraacetic acid (EDTA) in ultrapure distilled DNAse/RNase‐free water (ThermoFischer). The samples were incubated at 65°C in papain solution overnight. After successful digestion in papain, the samples were analyzed for dsDNA content using the Quant IT Picogreen assay (Thermo‐Scientific) as per the manufacturer's instructions. Fluorescence values were measured at ex 490 nm/em 510–570 using a Modulus II Microplate reader.

### 2.9 Albumin secretion

Secreted albumin was quantified using the Bromocresol Green Assay (BCG, Sigma). Media was changed on scaffolds 24 h prior, and media from *N* = 5 samples per group was preserved at −80°C before measurement. The assay was conducted as per manufacturer's instruction and absorbance at 570 nm was measured on a Modulus II Microplate Reader.

### 2.10 Immunohistochemical staining

4′,6‐diamidino‐2‐phenylindole (DAPI) and fluorescent conjugated Phalloidin‐514 staining was conducted to visualize the nuclei and f‐actin cytoskeletal filaments, respectively. Briefly, scaffolds were washed three times before permeabilisation with 0.2% Triton X‐100 in PBS. After permeabilisation, the scaffolds were then washed three times in PBS and stained with 300 nM DAPI solution in PBS for 10 min. After washing three times in PBS the scaffolds were stained with Phalloidin‐514 solution in PBS with 1% bovine serum albumin (BSA) for 30 min. The stained scaffolds were then washed 3 times and stored in PBS at 4°C awaiting imaging with a Zeiss Axio Imager II confocal epifluorescence microscope.

### 2.11 RT‐qPCR gene expression analysis

Scaffold bound cells from *N* = 5 samples per group were lysed immediately by placing entire scaffolds into Trizol reagent and stored at −80°C prior to RNA extraction. RNA extraction was conducted by addition of chloroform and centrifugation at 14,000*g* for 15 min. The clear RNA containing supernatant was extracted and washed on a Reliaprep Miniprep Spin Column (Promega) before elution with DEPC‐treated water. Yield RNA concentration was determined using a Nanodrop UV/Vis Spectrophotometer and concentrations were adjusted with DEPC water in preparation for cDNA production, samples were stored at −80°C. cDNA preparation was conducted using the Improm‐II Reverse Transcription kit (Promega) and stored at −20°C. qPCR was conducted on cDNA samples using the SYBR green dye reagent (Qiagen) with a Lightcycler 480 (Roche). Forward and reverse primer sequences used are available in the supplementary information.

### 2.12 Statistical analyses

Numerical results are presented as mean ± standard deviation (SD) unless otherwise stated. All grouped data was analyzed by one‐way ANOVA with Tukey's Post‐Hoc testing using the MATLAB scripting software, with comparisons between groups and timepoints.

## 3 RESULTS

### 3.1 Rat liver decellularization

Whole organ decellularisation by vascular perfusion was achieved in 32 h with the liver undergoing a complete color change from opaque brown to translucent white as seen in Figure 1 *.B*. Decellularisation was confirmed, shown in Figure 1 *.C* by a 96% drop in dsDNA content from 397.9 ± 86.7 ng/mg in tissue to 16.6 ± 2.5 ng/mg in rLECM, dry weight.

### 3.2 Scaffold fabrication

**FIGURE 2:**
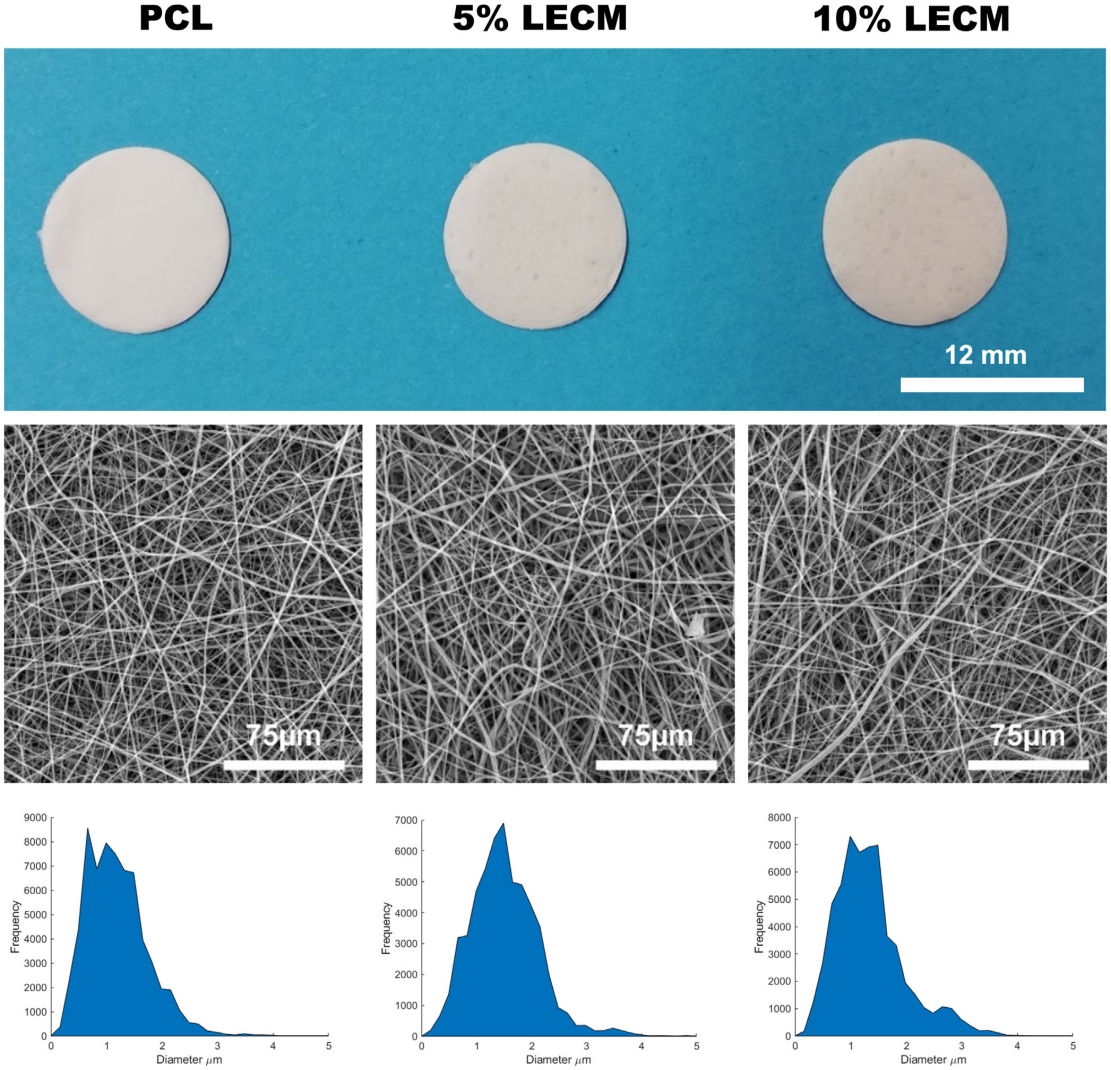
Gross images of the electrospun scaffolds (Top‐row). SEM images of the scaffold fibers (Middle‐row). DiameterJ fiber diameter histograms showing the distribution of fiber size within the scaffolds (Bottom‐row)

Fibers were fabricated by electrospinning, shown in Figure 2, and display similar randomly oriented fiber morphologies between each of the PCL, 5% rLECM and 10% rLECM groups. Measurements of fiber diameters using the DiameterJ software showed the average fiber diameters to be 1.07 ± 0.52 μm for PCL only, 1.45 ± 0.57 μm for 5% rLECM and 1.19 ± 0.49 μm for 10% rLECM. The spread of the histograms shown in Figure 2 shows that the fiber size distributions had similar characteristics between each of the groups. A speckled appearance is seen on the rLECM containing scaffolds where large rLECM particles have drawn drops of polymer solution.

### 3.3 Scaffold mechanical properties

**FIGURE 3:**
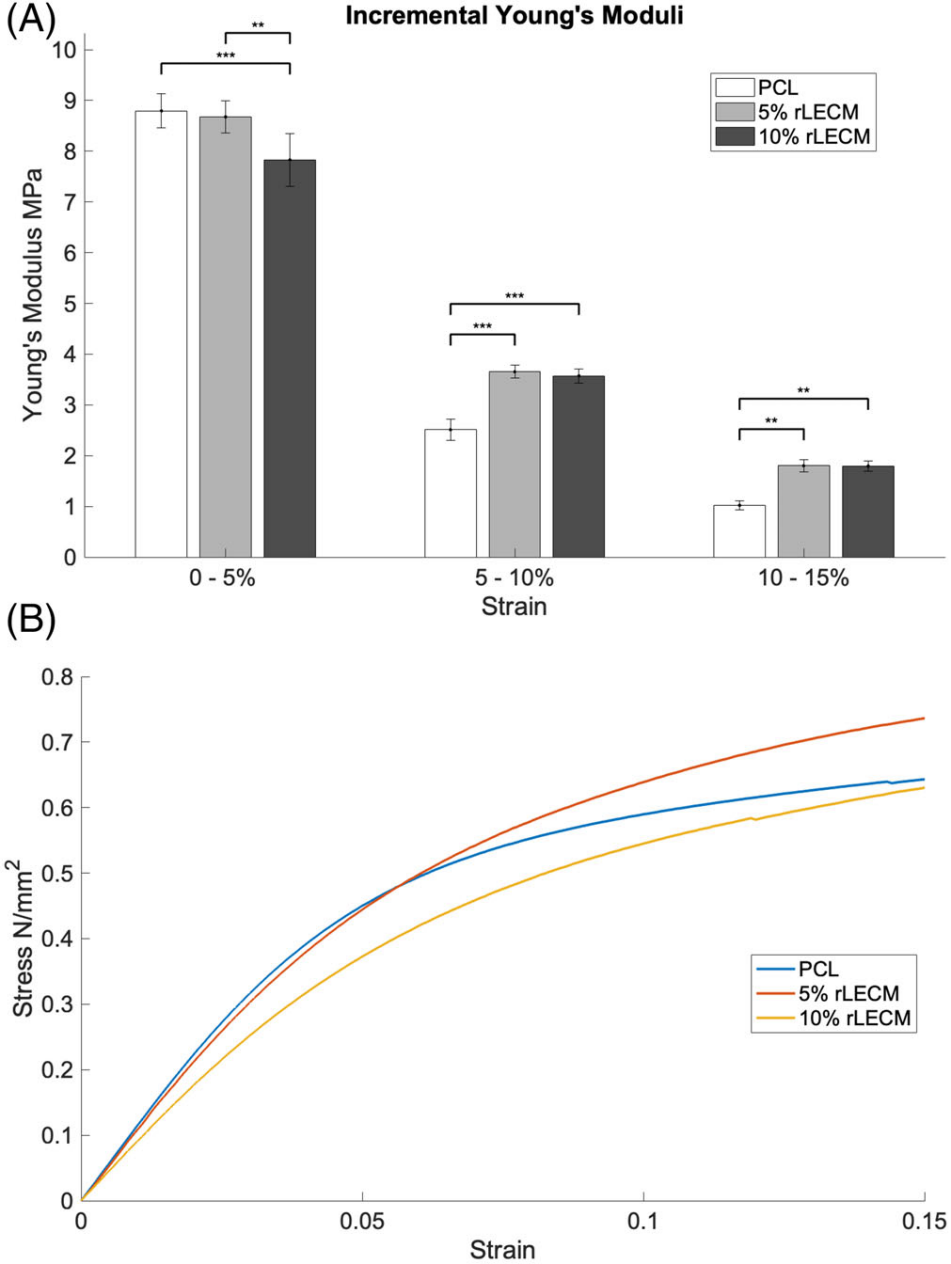
(A) Graph showing the incremental Young's moduli for each scaffold at strain increments 0%–5%, 5%–10%, 10%–15%. *N* = 5, Error bars = SD, One‐way ANOVA results with Tukey's Post‐hoc test shown. *p < .05; ** *p* < .01; and ***p < .001. B) Graph showing the stress/strain relationship for each scaffold material upon extension

Analysis of the tensile properties of each of the scaffold materials (Figure 3) between 0% and 5% strain revealed the 10% rLECM group to have a significantly reduced Young's Modulus in comparison to the PCL and 5% rLECM groups by 0.96 and 0.85 MPa, respectively. Notably, upon further extension, material stiffness dropped more sharply in the PCL only scaffold in comparison to the rLECM containing groups, as a result the Youngs modulus of PCL at 10%–15% strain is 0.78 and 0.77 MPa less than the 5% rLECM and 10% rLECM groups, respectively.

### 3.4 Scaffold FTIR and CN analysis

**FIGURE 4:**
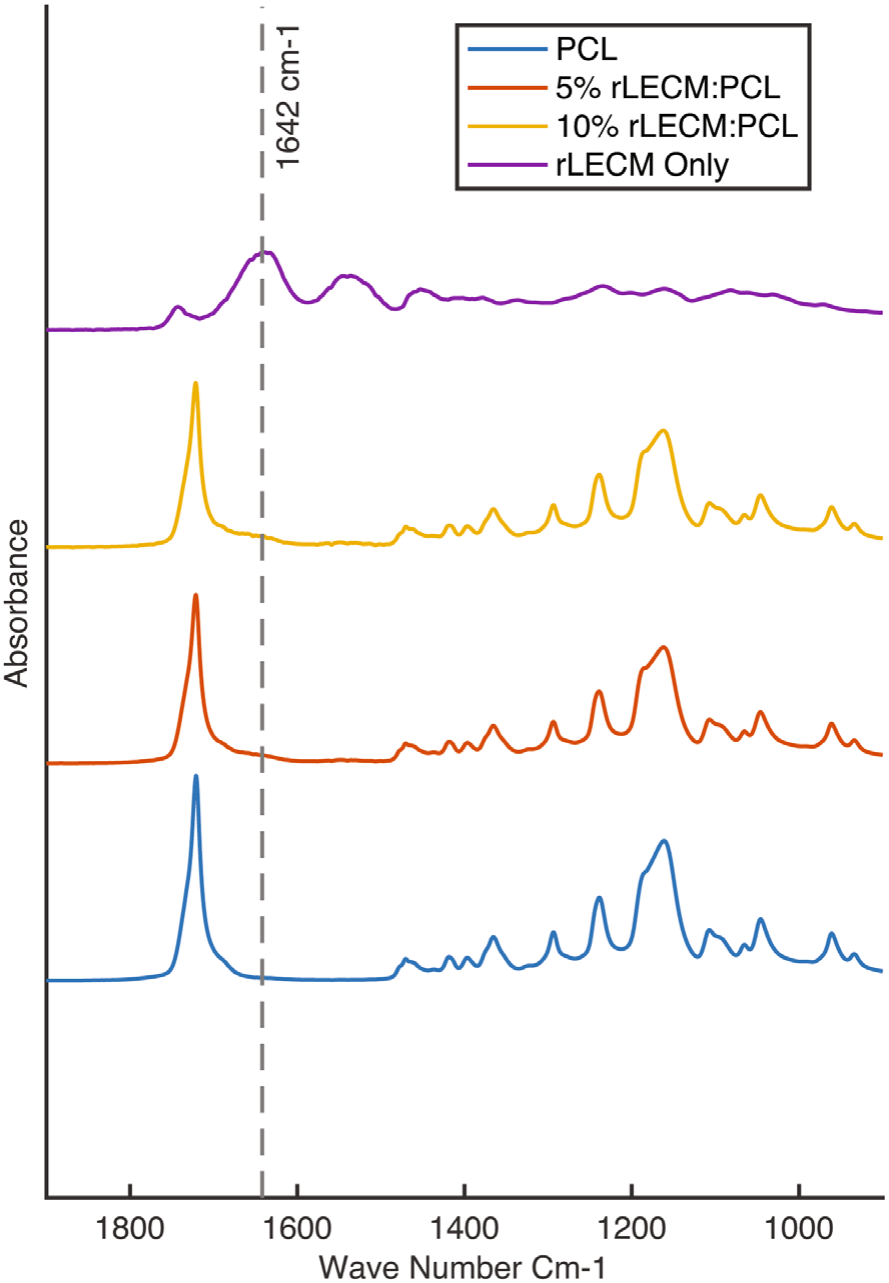
Graph showing FTIR absorbance spectra of rLECM, PCL, 5% rLECM:PCL and 10% rLECM:PCL. Dashed line marker at 1642 cm^−1^ shows the amide I vibrational characteristic band

FTIR absorbance spectra were obtained for rLECM powder, PCL fibers, 5% rLECM:PCL fibers and 10% rLECM:PCL fibers. The spectra, seen in Figure 4 show the Amide I characteristic vibrational band (1642 cm^−1^)^33^ present in the rLECM, 5% rLECM fibers and 10% rLECM fibers. The peak at 1642 cm^−1^ is absent in the absorbance spectra for PCL and the peaks for PCL are visible in the 5% rLECM fibers and 10% rLECM fibers.

Table 1 shows the absorbance values at the amide I band for the scaffold samples which increase with the addition of rLECM into the PCL. To confirm that this was due to protein addition, an elemental analysis was conducted to measure the percentage of nitrogen in the samples. This confirmed that the percentage of nitrogen within the scaffold materials increased with the addition of rLECM. A doubling of nitrogen would have been expected between the 5% rLECM and 10% rLECM samples; however, the 10% rLECM sample had 1.55 times the nitrogen compared with the 5% rLECM.

**TABLE 1:** Chemical analysis of the scaffold samples showing FTIR absorbance values at 1642 cm^−1^(amide I content) and CN analysis results showing the percentages of carbon and nitrogen within the scaffold materials, *N* = 3 and results displayed as Mean ± SD

	PCL	5% rLECM	10% rLECM
IR Abs @ 1642 cm^−1^	0.016	0.044	0.055
% Nitrogen	0.10 ± 0.01	0.60 ± 0.01	0.87 ± 0.01
% Carbon	63.21 ± 0.12	62.63 ± 0.10	62.42 ± 0.05

### 3.5 HepG2 attachment and proliferation

**FIGURE 5:**
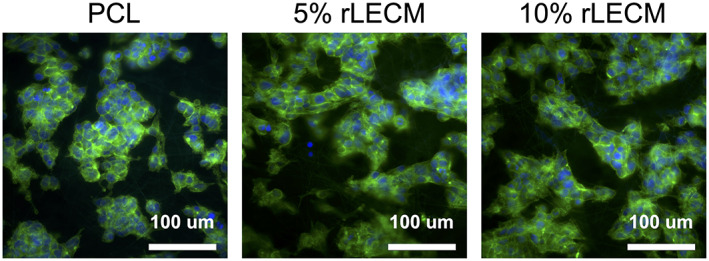
Images showing nuclei (DAPI) and f‐Actin filaments (Phalloidin) of HepG2 cells upon the PCL, 5% rLECM:PCL and 10% rLECM:PCL scaffolds at 48 h culture. Images taken with a 40X objective. DAPI = Blue, Phalloidin = Green

**FIGURE 6:**
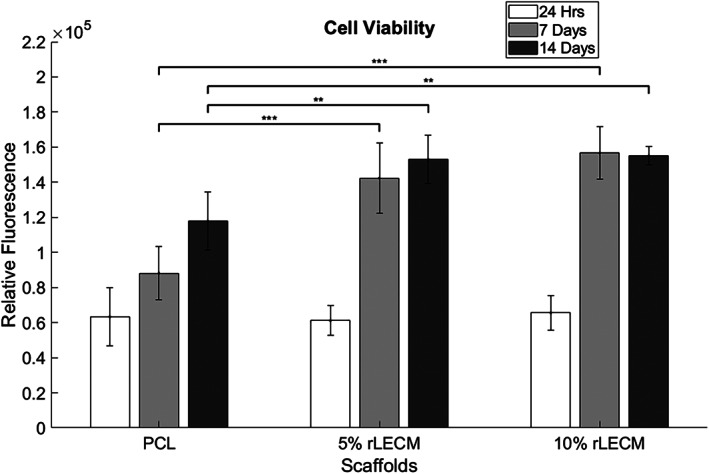
Graph showing cell viability data for HepG2 culture on each of the scaffolds at 24 h, 7 days and 14 days. *N* = 5, Error bars = SD, One‐way ANOVA results with Tukey's Post‐hoc test shown. *p < .05; **p < .01; and ***p < .001

HepG2 cells attached to all scaffold groups and the morphology of the scaffold bound cells can be observed in Figure 5. Over 14 days of culture cell viability increases on all scaffolds in accordance with normal proliferative behavior of HepG2 cells. At 7 and 14 days cell viability is significantly higher in both the 5% rLECM and 10% rLECM scaffolds compared with PCL only (*p* < .001 at 7 days and *p* < .01 at 14 days) (Figure  6).

**FIGURE 7:**
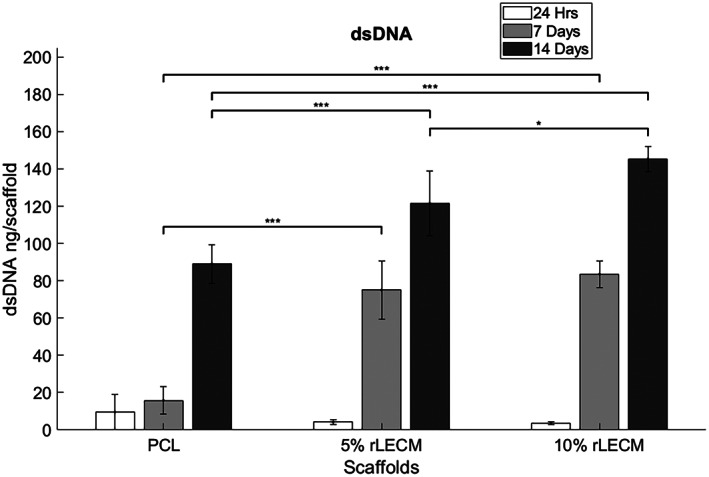
Graph showing the amounts of dsDNA in each of the HepG2 scaffold cultures at 24 h, 7 days and 14 days. *N* = 5, Error bars = SD, One‐way ANOVA results with Tukey's Post‐hoc test shown. *p < .05; **p < .01; and ***p < .001

Similar increasing trends were observed in the dsDNA content upon all the scaffolds over 14 days, seen in Figure 7. After 7 days the mean dsDNA on the PCL only scaffold increased by a factor of 1.6, while on the 5% rLECM and the 10% rLECM scaffolds the mean dsDNA increase was 17.9‐ and 27.9‐fold, respectively, presenting a significant increase in proliferation rates on rLECM containing scaffolds. After 14 days, the maximum dsDNA was held on the 10% rLECM scaffold with 145.3 ng followed by 5% rLECM at 121.4 ng and the PCL only scaffold with 89.0 ng.

### 3.6 Albumin secretion

**FIGURE 8:**
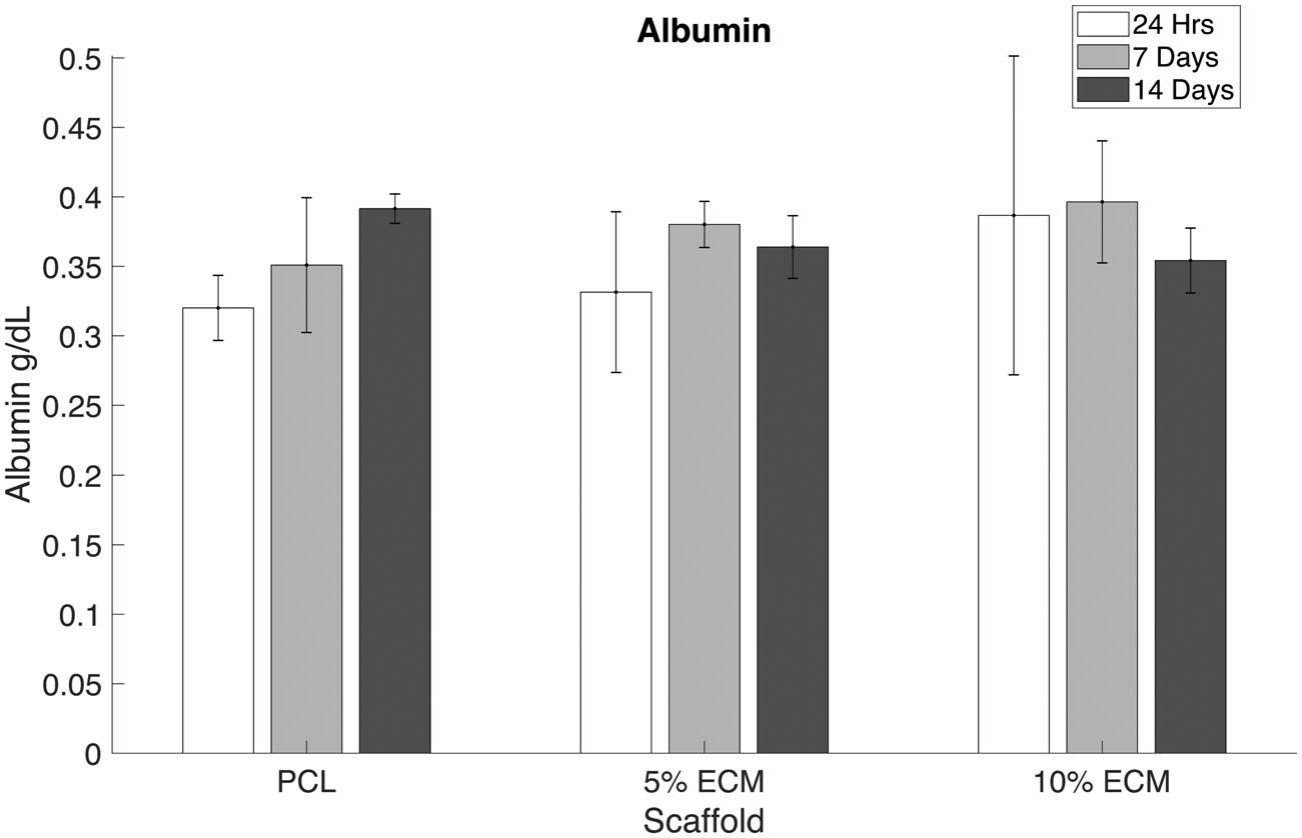
Bromocresol green results showing albumin concentration in the culture media after 24 h of HepG2 culture at 24 h, 7 days and 14 days. *N* = 5, Error bars = SD

Albumin levels within the culture media, shown in Figure 8, showed an increasing trend on the PCL only scaffold over 14 days. In the rLECM containing scaffolds there is an increasing concentration over the initial 7 days of culture, however, at 14 days the albumin concentration on the 5% rLECM and 10% rLECM scaffolds reduces by 0.01 and 0.04 g/dl, respectively.

### 3.7 Gene expression

**FIGURE 9:**
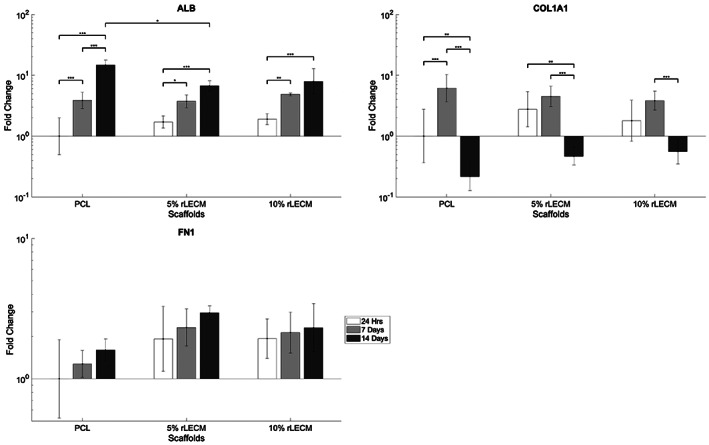
RT‐qPCR results showing Albumin, COL1A1 and FN1 gene expression in HepG2s on each of the scaffolds over a 14‐day culture period. Results normalized to reference gene GAPDH and expression of target on PCL at 24 h. *N* = 5, Error bars = SD, One‐way ANOVA results with Tukey's Post‐hoc test shown. *p < .05; **p < 0.01; and ***p < 0.001

RT‐qPCR analysis obtained relative gene expression data for the hepatic marker Albumin (ALB) and the ECM genes Col1a1 (Collagen I) and Fn1 (Fibronectin), shown in Figure 9. Albumin expression was seen to increase upon each scaffold over 14 days. A more rapid increase in albumin expression was observed upon the PCL only scaffold with a 14.6‐fold change in expression over 14 days, compared with 3.9 and 4.3 on the 5% rLECM and the 10% rLECM scaffolds, respectively. Notably, albumin expression was significantly higher at 14 days on the PCL only scaffold in comparison to the 5% rLECM containing scaffold (*p* < .05 PCL vs. 5% rLECM), a similar trend is observed on the 10% rLECM scaffold. Col1a1 expression follows a similar trend between each of the scaffold groups, with a more pronounced pattern demonstrated on the PCL only scaffold. Over 7 days Col1a1 expression increases 6.1 fold on the PCL only scaffold compared with 1.6 and 2.1 on the 5% rLECM and the 10% rLECM scaffolds respectively. From 7 to 14 days the decrease in Col1a1 expression is 29.2‐fold on the PCL only scaffold and on the 5% rLECM and 10% rLECM scaffolds it is 9.5‐ and 6.9‐fold, respectively. Fn1 expression exhibited an increasing trend upon each scaffold group, with expression marginally higher upon rLECM containing scaffolds. At 14 days Fn1 expression is 1.8 and 1.4 fold higher on the 5% rLECM and 10% rLECM scaffolds respectively, compared to the PCL only scaffold.

## 4 DISCUSSION

This study has demonstrated the utilization of rat liver ECM, obtained by whole organ vascular perfusion decellularisation, within electrospun PCL scaffolds. Our results suggest that the inclusion of rLECM establishes bioactive interactions between HepG2 cells and the scaffold, confirming the potential of using a controllable LECM source to introduce the hepatic microenvironment into electrospun PCL scaffolds.

Decellularisation of the entire rat liver was achieved over a 20‐h time period resulting in a whitish translucent material retaining the shape of the original liver tissue and showing an intact vasculature (see Figure 1B *)*. While some methods in literature have reported up to 72‐h decellularisation protocols, others report complete decellularisation at <5 h.^34^, ^35^, ^36^, ^37^, ^38^ It is likely that the relatively high time period (>5 h) required for complete decellularisation was due to canulation of the inferior vena cava. Most of the rat decellularisation protocols call for canulation of the hepatic artery or portal vein which provide well‐distributed flow through the vasculature by antegrade perfusion. To improve perfusate distribution in retrograde perfusion, the flow rate was increased to 10 ml/min in comparison to 5 ml/min commonly reported for antegrade perfusions.^34^, ^35^, ^36^, ^37^ Picogreen analysis of dsDNA content showed a reduction of 96% of the rat liver dsDNA by our decellularisation protocol, confirming successful removal of the majority of cellular components from the liver. A perfusate concentration of 0.1% SDS was chosen as it is known to effectively decellularise rat livers and has been shown also to preserve the integrity of collagens, glycosaminoglycans (GAGs) and other growth factor components of the decellularised ECM.^31^, ^34^, ^36^, ^37^, ^38^

The powdered rLECM obtained after ball milling was incorporated into 8% PCL:HFIP solutions at a ratio of 5 w/w% and 10 w/w% with respect to the PCL. The solutions were electrospun into scaffolds that were morphologically similar, with fiber diameters of 1.45 ± 0.57 μm for the 5% rLECM and 1.19 ± 0.49 μm for the 10% rLECM scaffolds. This, and morphological consistency with the PCL only control, allowed for the effects of rLECM to be assessed independent of morphological differences. As can be seen in Figure 5, the attached HepG2 cultures are morphologically similar between each of the scaffolds. The FTIR analysis confirmed that the incorporated protein material was present within the scaffolds and so this showed our method can successfully incorporate rLECM into electrospun scaffolds at different concentrations, while maintaining morphological consistency of the fibers. The FTIR and CN elemental analysis showed that the rLECM in the 10%.rLECM scaffold was not doubled compared with the 5% rLECM sample. This is likely due to both unavoidable inaccuracies associated with small batch processing of manufacturing materials, and losses of rLECM through the manufacturing process. Understanding where material losses are made will be important in order that ECM content can be controlled more precisely within the system. In Figure 2 it can be seen, particularly on the 10% rLECM, which a speckling of the material has formed during the electrospinning process. This was most probably due to large insoluble particles of the rLECM powder carrying a surrounding droplet of the polymer solution toward the collector. Though these defects are visible at a macroscale, the fibers observed within the speckles are not affected due to an overlay of fibers after the particle deposition. Reducing the likelihood of such defects forming would necessitate an analysis of the particle sizes of the rLECM after ball milling in order to confirm large particles as the causative factor.

Increases in cell viability and dsDNA content of the HepG2 cultures over 14 days indicated highly proliferative activity in accordance with normal HepG2 characteristics. Significantly increased levels of cell viability and dsDNA were observed on the rLECM containing scaffolds after 7 days and increasing with concentration of the rLECM. This is clear evidence that the incorporation of rLECM into the scaffolds has increased the rate of proliferation of the HepG2.

The proliferative activity shows an inverse correlation with the tensile elastic modulus of the scaffold material. The 10% rLECM scaffold shows a significant reduction in the Youngs' modulus between 0% and 5% strain which could be driving the differences seen in the proliferative activity of the HepG2. Reports in literature show that HepG2 proliferation tends to increase with increasing matrix stiffness, due to upregulation of cell cycle related proteins cyclin‐D1 and β‐catenin by mechanotransduction pathways.^39^, ^40^ The stiffness range of the substrates used in these experiments however did not cover the range observed between the scaffolds in our experiment. Thus, increased HepG2 proliferation upon the rLECM scaffolds cannot be conclusively attributed to the reducing tensile mechanical properties.

Discrepancies between the proliferation on the rLECM scaffolds compared with the PCL control also implies the possibility of retained functional cell attachment motifs and growth factor ligands within the scaffold material. It is known that ECM proteins can influence proliferation of HepG2 cells.^41^ For example, Collagen I promotes HepG2 proliferation via regulation of the Integrin β1/FAK signaling pathways.^42^ Decorin, another prominent ECM component, is understood to inhibit HepG2 proliferation by upregulation of TGF‐β1, which also has specific binding sites within the ECM for latent action.^43^, ^44^ However, HFIP is a solvent which has a high propensity for forming hydrogen bonds and so can disrupt and transform the secondary and tertiary structures of proteins.^45^, ^46^ HFIP has been used extensively to electrospin Collagens, though is argued that HFIP may partially transform the collagens toward gelatin‐like structures.^47^, ^48^ Thus, it is likely that the rLECM proteins within the electrospun scaffolds are structurally denatured to some degree by exposure to the HFIP. However, the demonstration of significantly altered proliferation of HepG2 on the rLECM containing scaffolds indicates that these structural protein damages do not totally impair the bioactive potential of the proteins. Further investigation into the interactions between cell surface receptors and scaffold bound ligands would be necessary to confirm if the preservation of protein functionality within the scaffolds is affecting the proliferative activity of the HepG2.

There are clear trends indicating that the rLECM content within the scaffolds have influenced the expression of genes within the HepG2 cultures. As can be seen from Figure 9 Albumin expression is significantly increased upon the PCL control compared with the rLECM containing scaffolds at 14 days. Furthermore, expression patterns of ECM genes and Col1a1 and Fn1 are distinct from the PCL control, though statistically significant differences were not observed. For all scaffold groups, there is a significant drop in Col1a1 expression over the 14 days of culture, indicating an eventual suppression of matrix remodeling processes associated with proliferating in‐vitro cultures. It has been shown in the previous studies that rLECM can alter the gene expression in HepG2 of many key functional proteins.^38^ Apart from rLECM content the significantly different cell numbers arising from the proliferation rates must be considered where cell number is known to affect the expression of certain proteins such as albumin from HepG2 cells.^49^ However, it cannot be deduced from the given data whether cell number is the driver of the differences observed between scaffolds or if it is due to interactions with the rLECM content of the scaffolds. Further characterization of the transcriptome and protein expression of the HepG2 cells would be necessary to confirm and analyze the effects of the scaffold materials on the hepG2 phenotype.

## 5 CONCLUSIONS

This study has demonstrated the successful incorporation of rat derived decellularised liver ECM into electrospun PCL scaffolds. The addition of rLECM into the PCL fibers stimulated a significantly increased proliferation rate in hepG2 cells, showing retention of bioactivity within the rLECM through the electrospinning process. Measured gene activity showed that the rLECM maintained key gene expression, with different trends observed over the 14‐day culture period. This presents a practical platform upon which hepatocytes can be cultured and raises questions regarding the preservation and bioactivity of rLECM within electrospun PCL scaffolds.

## Supporting information


**Figure S1** Graph showing the method for calculating the incremental young's modulus from the stress strain relationship of the scaffold materials.

## Data Availability

The data that support the findings of this study are available from the corresponding author upon reasonable request.
