# Supplementary material for: Rat liver ECM incorporated into electrospun polycaprolactone scaffolds as a platform for hepatocyte culture
Source: J Biomed Mater Res B Appl Biomater. 2022 Jun 23;110(12):2612–23. doi: 10.1002/jbm.b.35115 (PMC9796056; doi:10.1002/jbm.b.35115)
Supplement: Supplementary file 1 — Figure S1 Graph showing the method for calculating the incremental young's modulus from the stress strain relationship of the scaffold materials. [file JBM-110-2612-s001.docx]

Supplementary Figures

*
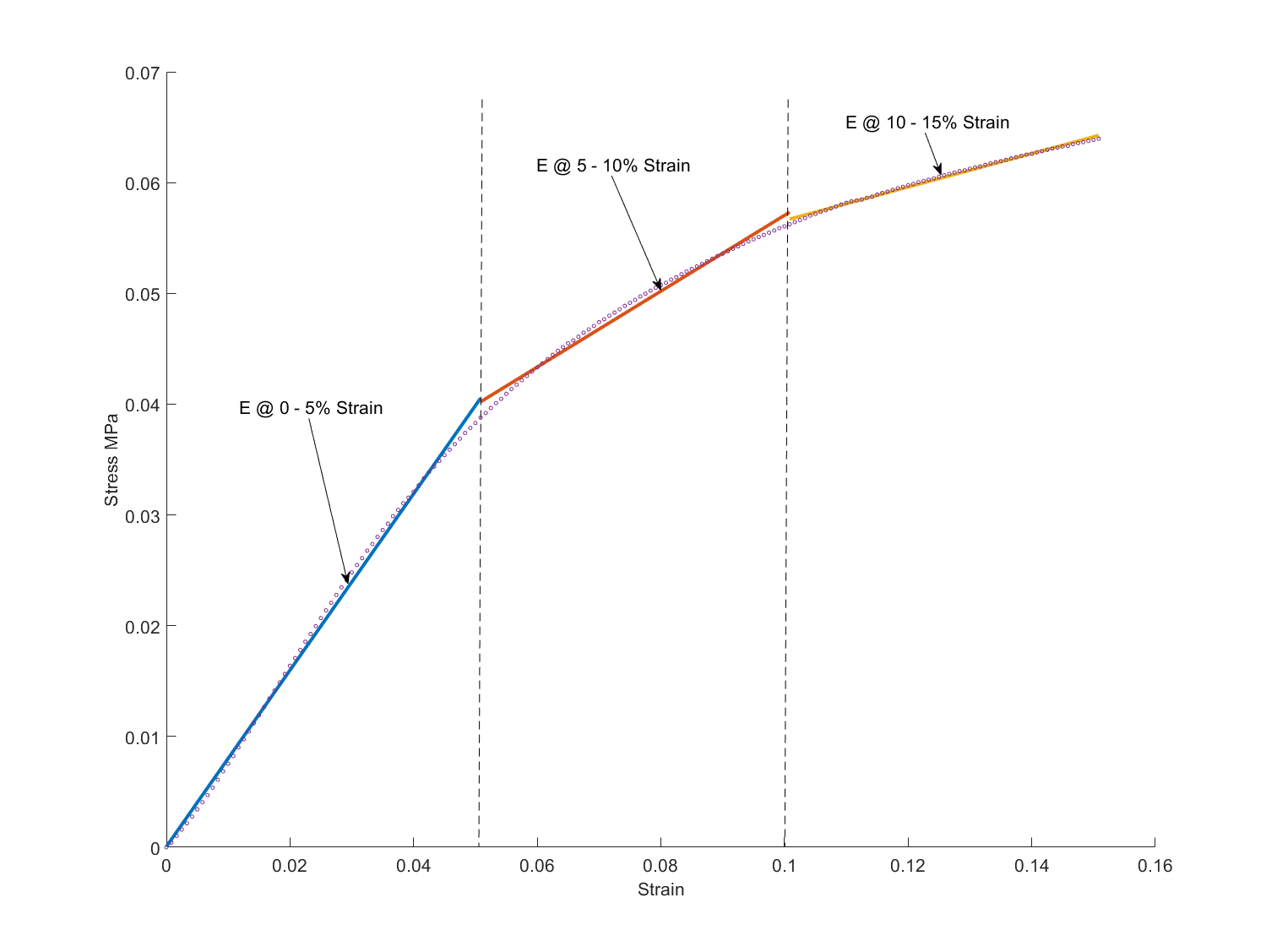
*

Figure 1: Graph showing the method for calculating the incremental young's modulus from the stress strain relationship of the scaffold materials.
